# Social and Mental Health Impact of Nuclear Disaster in Survivors: A Narrative Review

**DOI:** 10.3390/bs11080113

**Published:** 2021-08-23

**Authors:** Caley Longmuir, Vincent I. O. Agyapong

**Affiliations:** Department of Psychiatry, University of Alberta, Edmonton, AB T6G 2R3, Canada; agyapong@ualberta.ca

**Keywords:** radioactive hazard release, nuclear disaster, anxiety, depression, post-traumatic stress disorder, psychological resilience, community mental health services, health policy, post-disaster interventions

## Abstract

This narrative review synthesizes the literature on the psychological consequences of the Three Mile Island nuclear accident of 1979, the Chernobyl nuclear disaster of 1986, and the Fukushima nuclear disaster of 2011. A search was conducted on OVID for studies in English from 1966 to 2020. Fifty-nine studies were included. Living through a nuclear disaster is associated with higher levels of PTSD, depression, and anxiety. Decontamination workers, those living in closest proximity to the reactor, and evacuees experience higher rates of mental health problems after a nuclear disaster. Those with greater psychological resilience and social supports experience lower rates of psychological distress. Individual-level interventions, such as mindfulness training, behavioral activation, and cognitive reappraisal training, have shown modest benefits on improving psychological wellbeing. At the population level, many of the measures in place aimed at reducing exposure to radiation actually increase individuals’ anxiety. Technology-based supports have been studied in other types of natural disasters and it may be beneficial to look at mobile-based interventions for future nuclear disasters.

## 1. Introduction

Although nuclear disasters have been relatively uncommon throughout history, their psychological impact is long-lasting and widespread. This paper will describe the sequelae of mental health conditions related to nuclear disasters that led to actual or threat of radiation exposure. This paper includes data from the Three Mile Island accident of 1979, the Chernobyl disaster of 1986, and the Fukushima Daiichi nuclear disaster of 2011. This paper will not include exposure to radiation from medical means or nuclear warfare.

Research on the psychological impact of nuclear disasters began in the aftermath of the Three Mile Island accident, which occurred in Pennsylvania in 1979. This was a level 5 nuclear disaster on the International Nuclear Event Scale (INES). In the initial period after the accident, Three Mile Island residents received contradictory information about radiation exposure and an evacuation advisory was released for pregnant women and families with young children [1]. Although a radiation leak did occur from the plant, there has been no evidence to suggest that any residents of Three Mile Island were exposed to high enough levels of radiation to cause physiological consequences [1]. Nonetheless, the threat of radiation exposure still contributed to mental health distress in the residents of Three Mile Island [1,2,3,4,5,6,7].

The Chernobyl disaster was the first level 7 nuclear disaster in history and remains the biggest nuclear disaster to date. Despite the magnitude of this disaster, research on the Chernobyl nuclear disaster is limited as research coming out of the Soviet Union during this time period was restricted. Research on the physiological consequences of the Chernobyl nuclear disaster indicates several thousand thyroid cancer cases directly attributable to the disaster, increased prevalence of leukemia among decontamination workers, and 134 confirmed cases of acute radiation syndrome [8]. Approximately 50 people died as a result of high levels of acute radiation [8].

Research in the area of nuclear disasters proliferated exponentially in the aftermath of the Fukushima nuclear disaster of 2011. The Fukushima nuclear disaster was triggered by the Tohoku earthquake and tsunami. The earthquake, with a magnitude of 9.0, triggered an automatic shutdown of reactors and the subsequent tsunami flooded the nuclear power plant, which damaged the cooling system [8]. As with the Chernobyl nuclear disaster, the Fukushima nuclear disaster was classified as a level 7 nuclear disaster, but the evacuation area around the plant was much smaller and the health effects have been significantly lower [8]. Unlike Chernobyl, there were no deaths from acute radiation effects in Fukushima and no cases of acute radiation syndrome [8]. Despite this, 116,000 people had to be evacuated and many elderly and hospitalized people lost their lives in the evacuation process [9].

Though nuclear accidents are uncommon, they lead to serious physical and mental health issues. There is a large breadth of literature on the physical consequences of nuclear disasters and radiation exposure, but psychological sequelae have been less widely studied until recently. Research on the mental health consequences of the Fukushima nuclear disaster has recently been summarized by a two-part systematic review [10,11] and a systematic qualitative review [12]. These articles summarize the psychological consequences of nuclear disasters, including increased levels of general psychological distress, depressive symptoms, post-traumatic stress symptoms [10], and radiation anxiety [12], as well as behavioral consequences, such as increased suicide rates [11]. There have been no reviews to date on the mental health consequences of nuclear disasters that include studies from multiple nuclear accidents. For the purposes of this review, radiation anxiety will be defined as health anxiety due to perceived radiation exposure, actual radiation exposure, or potential for radiation exposure in the future. This definition is based on previous research [10,11,13,14,15,16,17] and includes concern about current health status, delayed health effects, and genetic effects on offspring and future generations.

This paper aims to outline the impact of nuclear disasters on mental health. The types of psychological sequelae that most commonly occur after living through a nuclear disaster, including symptoms of post-traumatic stress disorder, depression, and anxiety, will be reviewed and the risk factors and protective factors surrounding the development of these conditions will be described. Specific groups of people, such as plant workers, clean-up workers, and those residing closest to the nuclear reactor, will be discussed, as they have an increased risk of exposure to radioactive material. Recommendations for future research, as well as policies and programs to mitigate the risk of development of mental health conditions post-nuclear disaster and to increase protective factors, will be addressed.

## 2. Methods

### 2.1. Search Strategy

**Figure 1:**
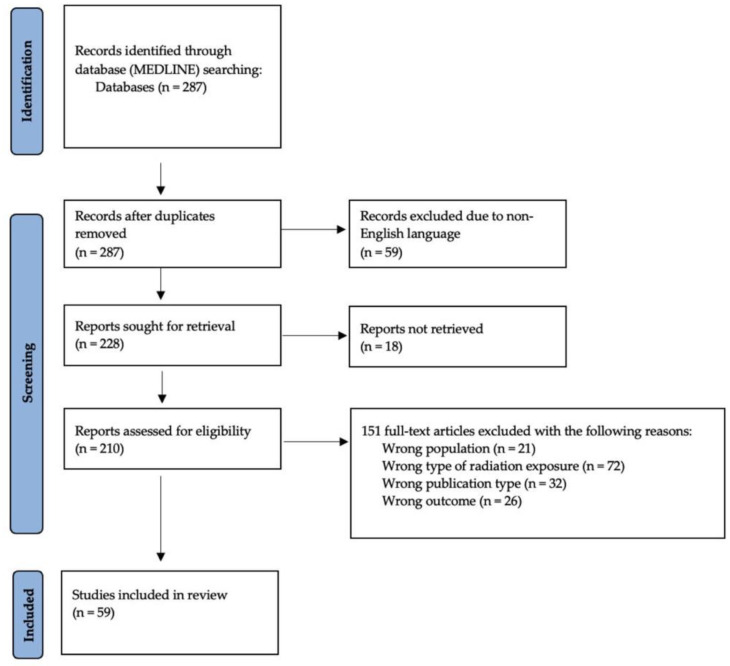
Flow diagram of article selection process, based on Page et al. (2020) [18].

A literature search of the MEDLINE database through OVID was conducted by one author (C.L.) in December 2020. The search used the MeSH terms “radioactive hazard release”, “nuclear reactors”, “radiation injuries”, “anxiety disorders”, “anxiety”, “depressive disorder”, “depression”, “dysthymic disorder”, “depression, reactive”, “adjustment disorders”, “suicide”, and “stress disorders, post-traumatic”. The stages of the literature search are presented in Figure 1. This search strategy yielded 287 research articles.

### 2.2. Inclusion and Exclusion Criteria

This review includes articles published in English between 1966 and 2020. We only included articles with a study population of adults who lived through a nuclear disaster. This excluded 11 studies on children, eight on animals, and two on people who did not experience a nuclear disaster firsthand. Thirty-four articles were excluded because of publication type. Editorials, letters to the editor, policy papers, case reports, news articles, conference presentations, and research studies that were purely qualitative in nature were excluded.

For inclusion in this review, the source of radiation in the study had to be from a nuclear disaster. This excluded 64 articles that pertained to radiation exposure from oncology treatments, medical imaging, and other types of medical radiation. We excluded three studies on radiation secondary to the atomic bombings of Hiroshima and Nagasaki in 1945 and one study on a radioactive contamination accident secondary to stolen radiotherapy equipment in Goiania in 1987. Four articles were excluding for studying microwave radiation, tanning beds, or mobile phone radiation.

We defined the outcome measures as “psychological consequences”, including, but not limited to, psychiatric diagnoses such as PTSD, depression, or anxiety, or clinically significant symptoms pertaining to these diagnoses. Two articles were excluded because they only studied acute stress in the peritraumatic period and did not address any other psychological consequences. We excluded studies with primarily physiological consequences as their outcomes. We also excluded studies that had “general psychological distress” as their only outcome measure pertaining to mental health. This further limited the search by 26 papers. After applying the inclusion and exclusion criteria to the search results, 59 studies were included in this review.

## 3. Results

Table 1, Table 2 and Table 3 summarize the key literature on the psychological consequences of nuclear disasters.

## 4. Discussion

The literature on the mental health consequences of nuclear disasters revealed increased prevalence of PTSD, depression, and anxiety. Each of these conditions has associated risk factors and will be discussed in turn. Vulnerable populations and protective factors will be identified because this can help policymakers know where to first allocate services in the aftermath of a nuclear disaster. Although there has been a limited amount of research on interventions aimed at mitigating psychological distress after a nuclear disaster, three interventions with modestly positive outlines will be discussed. Population-level interventions, such as radiation countermeasures and media strategies, are outlined. Technology-based supports, such as supportive text messages, that have been successful in the aftermath of other disasters are discussed. Significant limitations are discussed and suggestions for future research are provided.

## 5. Key Mental Health Disorders and Associated Risk Factors

### 5.1. Post-Traumatic Stress Disorder (PTSD)

Stress often peaks during disaster-related events, remains high for a period of time afterwards, and then, gradually decreases [22,26,35,53,55,60]. Lasting symptoms of stress can include hypervigilance, avoidance of reminders of the event, flashbacks, and nightmares. These symptoms may bother people for years after the traumatic incident. PTSD symptomatology rates range from 33.2 to 59.4% in the first year after experiencing a nuclear accident [44,62].

Not everyone who lives through a nuclear disaster is affected the same way. There are individual variables and disaster-related variables that play a role in the psychological outcomes. Individual variables, such as social isolation [62] and having a pre-existing physical or mental illness [59,62], were associated with higher levels of PTSD. People who experienced discrimination or slurs in the aftermath of a nuclear disaster had higher levels of post-traumatic stress [59,60] and a more prolonged course of post-traumatic stress response symptoms [35]. Concern about livelihood and lost jobs were also associated with PTSD [62].

Disaster-related variables, such as witnessing the plant exploding and experiencing life-threatening danger, were associated with a more prolonged course of post-traumatic stress response symptoms [35]. Higher levels of stress experienced at the time of the nuclear disaster, or in the immediate aftermath of the disaster, have been linked to higher levels of stress a year after the disaster [59,60].

Greater exposure to radiation was associated with greater PTSD symptoms both in the year after the accident and 18 years after the accident [25]. Even if there is no actual radiation exposure, living next to a nuclear reactor that has been experiencing problems leads to higher levels of stress than living next to a normally functioning nuclear plant [1,2]. People required to evacuate their homes due to a nuclear disaster are at a higher risk of developing post-traumatic stress response symptoms [35] and are more likely to fit the criteria for a formal PTSD diagnosis [20,21,53]. Evacuees face the compounded risk of greater exposure to radiation due to their location, the stressors of relocation [20], and fear of future nuclear events.

### 5.2. Depression and Suicidality

Depression is also more prevalent in the aftermath of a nuclear disaster. Studies have found that 21.1–66.8% of people experience depressive symptoms [37,44] and 7.1–23% of people meet criteria for a full diagnosis of depression [7,47,61] in the first year after a nuclear accident.

Disaster-related stressors that were associated with greater depressive symptoms were having to evacuate one’s home due to a nuclear disaster [20,42], income reduction, and home water incursion [47]. People with a history of psychiatric illness are also more likely to screen positive for depression in the aftermath of a nuclear disaster [4,31].

Proximity to the nuclear plant and radiation exposure are associated with depression. Those who lived closest to the nuclear reactor reported greater levels of depressive symptoms [37] and had higher rates of depression [61]. Clean-up workers in Chernobyl who experienced acute radiation sickness (ARS) were more likely to experience depressive symptoms than clean-up workers who did not experience ARS [19]. More specifically, a more significant received dose of external radiation exposure was associated with more depressive symptoms, a formal diagnosis of depression, and the severity of the depression [19]. More Chernobyl clean-up workers experienced depression and suicidal ideation after the accident than a control group of non-clean-up workers from the same area, and this pattern was consistent even 18 years after the incident [25].

Suicidal ideation is often discussed in the context of depression but has been less studied in the nuclear disaster literature. One study found that two years after the Fukushima nuclear accident, 8.9% of public employees were considered to have a “suicide risk” [49]. Chernobyl workers were more likely to report suicidal ideation than a similar group of non-clean-up workers from the Chernobyl area, with rates of 9.2% and 4.1%, respectively [25]. In addition to increased suicidal ideation, rates of suicide attempts and deaths by suicide were also impacted by the nuclear disaster. The risk of non-fatal suicide attempt via high mortality means (jumping from a significant height, hanging, or stabbing) was significantly higher for four months after the disaster and then decreased to baseline [29]. Rates of completed suicide were affected on a more long-term scale. Men from Estonia who participated in the Chernobyl clean-up had an increased risk of death by suicide compared to a control group from Estonia, but no increase in overall mortality rate 17 years after the accident [27]. In Fukushima, female suicide rates started increasing 1.5 years after the nuclear disaster and male suicide rates started increasing 2.5 years after the accident, although they had initially increased for a brief period immediately after the accident before returning to baseline [56]. For men, changes in suicide rate differed based on age group [56]. Suicide rates decreased for men 50–69 and increased for men younger than 30 and 70 and older [56].

### 5.3. Anxiety Disorders

In addition to anxiety-related to post-traumatic stress disorder and its associated symptoms, nuclear disasters can contribute to other types of anxiety. This can include generalized anxiety disorder, health anxiety, and non-specific anxious symptomatology. Anxiety surrounding radiation exposure and future health consequences from radiation exposure is a large focus of research on anxiety and nuclear disasters.

Damage to one’s home from the nuclear disaster and having to evacuate one’s home after the disaster are associated with more significant anxiety [13,21,42,57]. People evacuated to temporary housing had higher rates of generalized anxiety disorder [42]. Radiation anxiety was also shown to be higher in evacuees than non-evacuees [13,21,42].

Anxiety in the aftermath of a nuclear disaster differs from other disasters because of the ongoing threats to health that radiation exposure holds. Rather than the disaster being a discrete stressor, the stress is ongoing due to potential future risks from radiation exposure. Common concerns from radiation exposure are thyroid cancer and other types of cancer, concern about the next generation, food contamination, soil contamination, and genetic effects [43,51]. Those who perceive the risk of radiation exposure as higher have greater levels of psychological distress [4,13,21,46,50,57]. As time passes from the nuclear accident, concern about radiation decreases [32,43].

## 6. Protective Factors and Vulnerable Populations

Research has demonstrated several protective features for those who experience accidental radiation exposure. Resilience is a protective factor for depression and PTSD after a nuclear disaster [44]. Laughter has been shown to be a protective factor for Fukushima evacuees, as it is associated with improved psychological health [53] and lower perceptions of genetic risk [50]. Research has found that medical students who volunteered in the Fukushima relief efforts did not have detrimental mental health effects [28]. This could relate to a self-selection bias, because those who volunteer for post-disaster relief work may be less likely to experience mental health concerns [28], but it could also relate to volunteers’ satisfaction from being able to help in a traumatic situation rather than feeling helpless.

Social support is also a significant protective factor for those who experience a nuclear disaster [3,48,53]. For elderly people forced to evacuate to rental living conditions from the Fukushima disaster, engagement in social activities was a protective factor against the development of depression [45]. Greater social support reduced the likelihood of a diagnosis of major depressive disorder or generalized anxiety disorder after a nuclear disaster [7].

Certain vulnerable populations have been identified in the aftermath of a nuclear disaster. Although research has shown impact on areas near and far to the disaster location [38], people living closest to the reactor are disproportionally affected by a nuclear disaster [22,24]. People who are forced to evacuate their homes due to a nuclear disaster have higher levels of distress [13,20,21,26,32,35,42]. This could be due to multiple mechanisms, as evacuees are more likely to have had actual radiation exposure but are also more likely to suffer social consequences such as isolation and lost employment. People with pre-existing psychiatric conditions should also be considered as a group to monitor closely after a nuclear disaster [4].

Demographic variables, such as age and gender, may also affect how people are impacted by nuclear disasters. Most research indicates that women are at higher risk for PTSD [52,53], depression [42,45], and anxiety [57]. Other research has shown no association between gender and PTSD [62] or depression [47]. Older age tends to be associated with greater risk of adverse outcomes after living through a nuclear disaster [23,52,55,60], although not all research has shown this result [47,62]. Research has shown that older age is associated with a greater likelihood of PTSD [52,55,60], depression [45], and anxiety [33]. Although symptoms of depression and PTSD improved over time for all participants, they improved at a slower rate for people 55 and older [23]. Older age is also associated with other factors that predispose people to worse outcomes after a nuclear disaster. Many people emigrated out of the Soviet Union after the Chernobyl nuclear disaster and research has shown that immigrants aged 65 and older had more difficulty establishing social supports, finding employment, and learning the language [23]. Older decontamination workers were more likely to work in an unfamiliar environment and in inadequate working conditions [33]. All of these factors may predispose older adults to worse psychological outcomes. In contrast, one study found that younger people (age 20–39) had higher levels of depression than middle-aged and older adults [47]. Another study showed that age was not associated with an increased likelihood of a PTSD diagnosis in either direction [62].

## 7. Comparison of Three Nuclear Disasters

The Three Mile Island incident, the Chernobyl nuclear disaster, and the Fukushima nuclear disaster are the three largest and most well-studied nuclear disasters in history. Despite all three disasters involving the release of radioactive material, the events had many differences. The Three Mile Island incident was a level 5 nuclear disaster on the International Nuclear Event Scale, indicating an accident with wider consequences, whereas Chernobyl and Fukushima were both level 7 nuclear disasters, indicating a major accident. Three Mile Island residents were not exposed to levels of radiation high enough to cause physical damage and had only a brief and voluntary evacuation warning [4]. No deaths have been attributed to the Three Mile Island incident [1]. In contrast, the Chernobyl nuclear disaster had both deaths from acute radiation exposure and delayed deaths from radiation exposure [8]. The Fukushima nuclear disaster caused no deaths from acute radiation [8], but deaths did occur in the context of the larger disaster, the Tohoku earthquake and tsunami. Fukushima had a much smaller evacuation zone than Chernobyl, more successful decontamination efforts, and significantly less health effects secondary to radiation exposure [8]. Despite both being level 7 nuclear disasters, some of the differences in outcome could be reasonably attributed to learning from the mistakes of Chernobyl.

All three of the nuclear disasters are associated with adverse psychological outcomes. Each of the nuclear disasters were associated with increased symptoms of PTSD [1,2,25,34] and depression [25,27,45] when compared to a control group. Suicidal ideation, attempts, and completed suicides increased in the aftermath of Chernobyl and Fukushima [25,27]. The rates of psychological sequelae are not comparable between disasters for multiple reasons. First, much of the research on the Chernobyl nuclear disaster was conducted 11–18 years after the accident. Second, the types of control groups used in the research varied between disasters. For the Three Mile Island incident, researchers compared the residents of Three Mile Island with people who lived near normally functioning nuclear plants [1,2]. The studies on Chernobyl use decontamination workers sent to Chernobyl from other countries compared to people from the same country who were not deployed [25,27]. The studies on Fukushima compare the rates of illnesses and symptoms in the same community pre- and post-nuclear disaster [29,34].

Certain factors made people more susceptible to experiencing adverse psychological outcomes in the aftermath of a nuclear disaster. Lack of social support was associated with adverse psychological outcomes for all three nuclear disasters [3,7,23,33,45,48,62]. Certain risk factors were unique to Chernobyl and Fukushima, given that the Three Mile Island nuclear incident did not release large quantities of radiation, require decontamination workers, or have a mandatory evacuation for residents [1]. For Chernobyl and Fukushima, living closer to the reactor, engaging in work with radiation exposure, and having to evacuate one’s home were risk factors for PTSD and depression [19,20,21,22,24,31,42].

One might hypothesize that Fukushima would have unique risk factors or outcomes compared to the other two nuclear disasters because of the co-occurring earthquake and tsunami, but the literature did not reflect this. The only significant risk factor that was found in Fukushima, but not the other nuclear disasters, was experiencing discrimination [59,60]. The Chernobyl nuclear disaster was caused by human error, whereas the nuclear meltdown in Fukushima was secondary to a natural disaster, yet nuclear plant workers in Fukushima still faced discrimination [59,60]. Discrimination was not a variable in the studies on Chernobyl or Three Mile Island included in this review. Only studies on the Fukushima nuclear disaster addressed protective factors and psychological interventions. This may reflect the large number of years that passed between Chernobyl and Fukushima and the advances in mental health research and treatment that occurred in that period.

## 8. Individual-Level Interventions Aimed at Mitigating Psychological Distress

People who experience nuclear disasters are more likely to struggle with lasting post-traumatic stress, depression, and anxiety. Measures and programs aimed towards mitigating the physical consequences of radiation exposure have been well-studied, but research on programs to improve psychological outcomes after a nuclear disaster has been scarce. Despite the availability of community mental health supports after a disaster, rates of service utilization have been low. In psychiatric patients, experiencing the Three Mile Island accident did not increase inpatient or outpatient service use [3]. Despite the risk of severe mental health sequelae, only 6% of nuclear plant workers from Fukushima had more than three mental health visits in the three years after the disaster [35].

Although research is limited due to the low number of nuclear disasters that have occurred in history, specific psychological interventions have been shown to be helpful in mitigating some of the negative mental health consequences of living through a nuclear disaster. These cognitive interventions include mindfulness training, behavioural activation, and cognitive reappraisal training [30,36,41]. A cross-sectional study based on online self-report questionnaires found that mindfulness has been associated with lower health anxiety and psychological distress, but not radiation risk perception [41]. This indicates that although people still perceive the same risks from radiation exposure, mindfulness training may decrease somatic symptoms by increasing one’s awareness of bodily sensations [41]. A randomized control trial of a two-session behavioural activation intervention was shown to have a small but significant impact on life satisfaction and livelihood, and a more intensive program could potentially have greater efficacy [36]. Learning to successfully use cognitive reappraisal skills to reduce negative emotions and thoughts associated with disaster-related pictures is associated with fewer symptoms of depression and PTSD in a correlational self-report study [30]. If people are able to re-evaluate how they think about the traumatic event, they may be able to reduce emotional reactivity, which is associated with poorer functioning [30]. Those who tend to benefit most from cognitive interventions are educated, employed, and have multiple children [36]. These three psychological interventions were studied in the aftermath of the Fukushima nuclear disaster and provide a basis for types of interventions that could be implemented in the aftermath of future nuclear disasters.

## 9. Population-Based Interventions, Public Policy, and Practice Interventions

### 9.1. Trust in Experts and Sources of Information

Where people seek information post-nuclear disaster and which sources of information are considered the most trustworthy can have an impact on mental health sequelae. After the Fukushima nuclear disaster, the Japanese government and the Tokyo Electric Power Company (TEPCO) were rated as low in credibility [43,57]. People who utilized the government as their main source of information had higher levels of anxiety [57]. People who reported a loss of faith in experts after a nuclear disaster had higher levels of psychological distress [5], anxiety, and depression [51]. People tended to rate mass media information sources as more reliable than government information [17], and thus, local media was utilized as a source of information more often than public relations information from the local government [16]. Improving the credibility of government information and reducing uncertainty is essential for mitigating the psychological impact of radiologic disasters [57]. Policies aimed towards bolstering trust in media and government sources of information may be beneficial.

Online sources of information have been examined for their associations with mental health sequelae. Some studies have found that utilizing internet sites and blogs as sources of information was associated with higher radiation anxiety [15]. Other studies found no evidence that social media was associated with anxiety about radiation risk [17]. This may indicate that anxiety is related to the type of information utilized online rather than the online form of media itself.

In-person sources have also been investigated for perceptions of trustworthiness. People rated information from family physicians and lectures held by radiation experts as the most reliable sources of information, more than any of the media or government sources [43]. Researchers suggested that this finding could be because these people are considered experts in the field of health and radiation or because in-person communication may have a greater impact on perceived trustworthiness than mass media communications [43]. Participation in a seminar on radiation health led to decreased anxiety about radiation risk [17]. Other in-person sources of information, such as citizen groups, word of mouth, and rumours, were associated with higher anxiety [15,17]. This indicates that it is likely the source of information rather than the in-person nature of the communication that is key to reducing psychological distress.

### 9.2. Radiation Countermeasures

Radiation countermeasures are measures implemented at the population level after a nuclear disaster to help mitigate the negative health implications of radiation exposure. The first radiation countermeasure implemented after a nuclear disaster is deploying decontamination workers to the areas with the highest radiation levels. When people evaluated the decontamination efforts of their town as successful, they reported lower radiation anxiety [14]. Unfortunately, the decontamination workers themselves face higher levels of radiation exposure and more significant psychological consequences, including PTSD, depression, and anxiety [19,25,27,33]. Specific measures must be taken to try to reduce the psychological impact of this type of work. Interventions such as training sessions, self-study materials, and wearing a mask have not been shown to decrease anxiety in decontamination workers [33]. This points to a critical area of future research.

In addition to widespread decontamination work, other radiation countermeasures are implemented to try to limit the negative impact of radiation on community members. Tools to measure an individual’s current level of radiation include whole-body counts, which are a measure of internal radiation, and individual dosimeters, which are a measure of external radiation [15]. Although aimed at preventing further radiation exposure, utilization of these particular radiation countermeasures was associated with higher levels of anxiety [15,51]. Attending explanatory meetings about radiation and paying close attention to radiation levels in food were also associated with higher levels of anxiety [15,51]. This increase in anxiety could be due to the countermeasures making the thought of radiation toxicity more salient in people’s minds, or it could be a selection bias that people who are already more anxious about radiation seek out and utilize these countermeasures. Although the aim of these countermeasures is to improve both physical and mental health, they instead might point us to a group of people who would benefit from further psychological interventions to reduce their distress. An important area of future research could focus on how to implement these types of community-wide programs without an increase in anxiety from participation in the radiation countermeasures.

### 9.3. Technology-Based Population Supports

Technology is ubiquitous in most developed countries today and may provide an effective way to reach people struggling with mental health concerns after living through a disaster. Research has shown that mobile phone-based population interventions are a cost-effective and valuable way to provide accessible psychological support [63,64,65,66,67,68,69,70,71]. These types of programs have been shown to decrease stress, depression, anxiety, and alcohol abuse [64,66,67,69,70,71]. A randomized control trial on psychiatric patients from Dublin in 2011 with dual diagnoses of depression and alcohol use disorder showed significantly reduced depressive symptoms and significantly greater abstinence from alcohol in the intervention group that received daily supportive text messages for three months compared to a control group that did not receive these messages [67]. Subsequent research found similar initial results but no lasting benefits six months after the cessation of the daily messages [70].

This type of mobile intervention has also been studied in remote populations. A mobile support program was effective in reducing depressive symptoms in Fort McMurry, Alberta, Canada, during the severe wildfires of 2016 [69]. This randomized control trial found that Fort McMurry residents diagnosed with Major Depressive Disorder who were assigned to the intervention group and received twice-daily supportive text messages for three months reported significantly lower depression scores on the Beck Depression Inventory than the control group (20.8 vs. 24.9) [69]. This program came to be known as Text4Mood, and this program was recognized as a mental health innovation by the Mental Health Innovations Network [71]. These types of mobile health interventions are useful in underserviced and remote areas where access to mental health services may be scarce or costly.

More recently, a similar program called Text4Hope has been developed and studied in Alberta, Canada. The goal of this program is to reduce psychological distress related to the COVID-19 pandemic and to promote resilience [63,64,65,66,68,71]. Text4Hope was created based on the Text4Mood mobile support program and provides subscribers with daily messages based on cognitive behavioural therapy [65]. The program was launched in March 2020, and within one week of launch, 32,805 Alberta residents had signed up for Text4Hope, indicating widespread uptake [63]. Demographic data indicate that people who self-subscribe to this program are mostly female (88%) and have an average age of 44.58 [71]. The average overall satisfaction with this program on a scale of 0–10 was 8.55 [71]. Most participants reported that the daily texts helped them cope with stress (77.1%), helped them cope with anxiety (75.8%), helped them feel connected to a support system (81%), helped them cope with COVID-related stressors (74%), and improved their mental well-being (75.6%). Two studies looking at stress measured with the Perceived Stress Scale-10 (PSS-10), anxiety measured with the General Anxiety Disorder Scale 7 (GAD-7), and depression measured with the Patient Health Questionnaire (PHQ-9) found decreased scores on all three scales in the intervention group who received the daily supportive messages compared to the control group [64,66]. Although these types of technologically based interventions have not been studied in prior nuclear disasters, they could be extremely useful to implement in the aftermath of a nuclear disaster, as they are able to be delivered remotely and would be accessible to those forced to evacuate because of the disaster.

## 10. Limitations

There were several limitations of this review. First, the majority of the studies used self-rating questionnaires to investigate symptoms of PTSD, depression, and anxiety, which are inferior to a clinical diagnosis [53]. Second, the most heavily studied nuclear disaster is the Fukushima nuclear disaster of 2011, which occurred in the wake of the Tohoku earthquake and tsunami, meaning that many people in the area experienced the stress of more than one type of disaster. Although this paper excluded studies that focused solely on the tsunami or earthquake, the effects of these disasters could not be controlled for and may have impacted those who experienced the nuclear disaster. Third, there are some limitations that are inherent to studying nuclear disasters, including both the difficulty finding ‘healthy controls’ sharing the same situation and the challenges in doing a pre–post design. Fourth, none of the reviewed literature addressed the role of pharmacology in the treatment of psychiatric conditions associated with nuclear disasters. Fifth, there are limitations inherent to qualitative narrative reviews. Narrative reviews are more subjective than systematic reviews. We attempted to mitigate this bias by outlining our search strategy and clearly stating our study inclusion criteria. Given the qualitive nature of this review, the goal was not to analyze the selected studies, but to synthesize the available literature. A relatively small sample size of 59 studies was included in this review. We chose to exclude manual searching to prioritize transparency in our study selection, but this may have limited the sample size by inadvertently excluding gray literature. Sixth, the studies addressing radiation exposure level did not use actual radiation measurements. They instead approximated higher or lower radiation exposure groups based on location of residence [22,23,24,31], evacuee status [13,16,26,49], or employment [19,23,25,26,33,52,59]. The lack of research on individual doses of radiation exposure and mental health outcomes makes it difficult to determine whether the symptoms are from the physiological impact radiation has on the brain or from the significant stress surrounding the event, which is also highest for those living the closest to the reactor, those required to evacuate, and those working in the highest risk jobs, such as nuclear plant workers at the time of the accident and decontamination workers after the accident.

## 11. Conclusions and Future Research

This review summarizes the adverse psychological outcomes associated with living through a nuclear disaster. The synthesis of studies from Three Mile Island, Chernobyl, and Fukushima nuclear disasters, indicate that survivors have higher levels of PTSD, depression, and anxiety than people who did not experience a nuclear disaster. Certain groups are disproportionally impacted by mental health sequelae after a nuclear disaster, including evacuees and those living in closest proximity to the nuclear reactor. Although the rates of each of these psychiatric conditions decrease over time since the nuclear incident, the significant impact these have on individuals and society should not be overlooked.

There are psychological interventions that have shown modest benefit in reducing the adverse psychological outcomes of nuclear disasters, including mindfulness training, behavioral activation, and cognitive reappraisal training. Research into these types of interventions in the aftermath of a nuclear disaster has been scarce; thus, further research in this area would be beneficial prior to the next large-scale nuclear disaster. Government-level interventions providing the public with credible sources of information in the aftermath of a nuclear disaster reduce fear surrounding radiation exposure. Although necessary, some of the measures that are put in place to mitigate the risk of radiation exposure in affected areas actually raise levels of mental health distress. Research could be carried out to see if there are any effective strategies to mitigate the rise in psychological distress due to the necessary radiation countermeasures. Suggestions for future research include technology-based interventions, such as mobile support programs, which are cost-effective strategies to reach large populations in geographically distributed areas.

## Figures and Tables

**Table 1:** Studies assessing psychological consequences of the Three Mile Island nuclear disaster.

Reference/ Nuclear Disaster	Sample	Final Sample Size	Study Period	Outcomes	Key Findings
Baum et al. (1983) [2]	Residents of Three Mile Island and three control groups	121 (38 in TMI group, 32 in undamaged nuclear plant group, 24 in coal plant group, 27 in no plant group)	August 1980	Stress (psychological, behavioural, and biochemical measures)	Residents of TMI exhibited more symptoms of stress (self-report, performance, and catecholamine levels) than the other three groups.
Bromet et al. (1982) [3]	Psychiatric patients treated in the six months prior to the Three Mile Island accident	215 (151 from Three Mile Island, 64 from comparison area)	December 1979–January 1980, March–April 1980	Mental health diagnoses (SADS-L), general psychological distress (GSI)	Rates of psychiatric conditions did not increase after the TMI accident. Greater psychological distress was associated with lower social support and perceiving the reactor as dangerous.
Davidson and Baum (1986) [1]	Residents living within 5 miles of Three Mile Island and a control group of residents living at least 80 miles from TMI	87 (52 in TMI group, 35 in control group)	January 1984	Stress (psychological, behavioral, and biochemical measures), PTSS (IES)	Residents of TMI exhibited more symptoms of stress (self-report, performance, and catecholamine levels) and greater PTSS.
Dew et al. (1987) [4]	Married women who delivered a child between January 1978 and March 1979 who experienced a community-wide stressor	361 (257 who lived within 10 miles of the TMI facility, 104 who lived near a plant that experienced widespread layoffs)	December 1979, March 1980, September 1981, September 1982, September 1983 (layoff group only)	Subclinical psychological symptomatology (SCL-90)	Levels of psychological symptoms were similar between groups at all timepoints. Presence of a pre-existing psychiatric diagnosis predicted enduring distress in both groups.
Prince-Embury and Rooney (1988) [5]	Residents of Three Mile Island at the time of the reactor restart in 1985	108	November 1985	Psychological symptoms (SCL-90-R)	Psychological symptoms were chronically elevated for residents who remained at TMI after the 1979 accident.
Prince-Embury and Rooney (1995) [6]	Residents of Three Mile Island at the time of the reactor restart in 1985 still living in the area in 1989	64	November 1985, June 1989	Psychological symptoms (SCL-90-R)	A lowering of psychological symptoms occurred between 1985 and 1989 despite increased lack of control, less faith in experts, and increased fear of developing cancer.
Solomon (1985) [7]	Mothers from two semi-rural regions of Pennsylvania (TMI and control)	436 (312 from Three Mile Island, 124 from the control area)	March–April 1980	Psychiatric disorder (SADS-L)	Women with worse social support were more likely to develop a psychiatric disorder following the TMI nuclear accident.

Abbreviations: GSI, Global Severity Index; IES, Impact of Event Scale; PTSS, post-traumatic stress symptoms; SADS-L, Schedule for Affective Disorders and Schizophrenia-Lifetime version; SCL-90, Symptom Checklist-90; SCL-90-R, Symptom Checklist-90-Revised; TMI, Three Mile Island.

**Table 2:** Studies assessing psychological consequences of the Chernobyl nuclear disaster.

Reference/ Nuclear Disaster	Sample	Final Sample Size	Study Period	Outcomes	Key Findings
Abramenko et al. (2017) [19]	Male clean-up workers	59	1986	Depressive symptoms	Workers who experienced ARS reported more depressive symptoms than those who did not.
Adams et al. (2011) [20]	Mothers with small children in Kyiv, Ukraine	797 (254 evacuees, 239 neighborhood controls, 203 population-based controls)	2005–2006	PTSD (IES-R), MDE (CIDI), general psychological distress (SCL-90)	Evacuees reported more negative risk perceptions and poorer overall well-being than the two control groups.
Bromet et al. (2002) [21]	Mothers with small children in Kyiv, Ukraine	600 (300 evacuees, 300 controls)	February–May 1997	Perceived health, Chernobyl-related stress, PTSD (IES)	Evacuees had worse health, more Chernobyl-related illness, higher Chernobyl-related stress, and greater rates of PTSD (18% of evacuees vs. 9.7% of controls).
Cwikel et al. (1997) [22]	Immigrants from the Commonwealth of Independent States (CIS) living in Israel	520 (87 from high-exposure areas, 217 from low-exposure areas, and 216 from comparison areas)	1993–1996	PTSS (IES), depression (CES-D), somatization (SCL-90), anxiety (SCL-90	At eight years after the accident, the exposure group had higher rates of PTSS, depressive symptoms, somatization, and anxiety than the comparison group.
Cwikel and Rozovski (1998) [23]	Immigrants from the Commonwealth ofIndependent States (CIS) living in Israel	520 (87 from high-exposure areas, 217 from low-exposure areas, and 216 from comparison areas)	1993–1996	PTSS (IES), depressive symptoms (CES-D), somatization (SCL-90), anxiety (SCL-90	Rates of somatization, depressive symptoms, and PTSS symptoms improved at a slower rate for immigrants who were 55 and older compared to younger immigrants.
Foster (2002) [24]	Russian immigrants residing in New York City	261	2001	Depression (BDI), anxiety (BAI), PTSS (MISS PTSD)	Participants who lived closer to the reactor had higher levels of anxiety and PTSS 15 years after the accident than those who lived further away.
Loganovsky et al. (2008) [25]	Male clean-up workers sent to Chernobyl between 1986 and 1990 and geographically matched controls	692 (295 clean-up workers, 397 geographically matched controls)	March–December 2002, December 2003–June 2004	Depressive disorders, anxiety disorders, alcohol abuse, and intermittent explosive disorder (CIDI), PTSD (IES), somatization (SCL-90), suicidal ideation	Clean-up workers were more likely than controls to experience depression (18.0% vs. 13.1%) and suicidal ideation (9.2% vs. 4.1%) after the Chernobyl accident. Eighteen years after the accident, rates of depression and PTSD were still elevated in the clean-up workers compared to the control group.
Loganovsky et al. (2013) [26]	Patients with PTSD and population controls	241 (34 Chernobyl clean-up workers with PTSD and ARS, 81 Chernobyl clean-up workers with PTSD without ARS, 76 Chernobyl evacuees with PTSD, 28 Afghanistan war veterans with PTSD, and 22 healthy controls without PTSD)	2011–2012	Radiation PTSD, neurological deficits, cognitive functions, neurophysiologic studies (EEG and carotid and cerebral ultrasounds)	Radiation PTSD includes “flashforward” phenomena, somatoform disorders, and neurocognitive deficits. Structural brain changes were demonstrated in Chernobyl clean-up workers, and changes in bioelectrical brain activity were demonstrated in Chernobyl survivors with PTSD.
Rahu et al. (2006) [27]	Men from Estonia who participated in the Chernobyl clean-up between 1986 and 1991	4786	1992–2002	Mortality	Compared to population rates, clean-up workers had increased risk of suicide, but no elevated mortality risk.

Abbreviations: ARS, acute radiation sickness; BAI, Beck Anxiety Inventory; BDI, Beck Depression Inventory; CES-D, Center for Epidemiologic Studies Depression Scale; CIDI, Composite International Diagnostic Interview; IES, Impact of Event Scale; IES-R, Impact of Event Scale-Revised; MDE, major depressive episode; MISS PTSD, Mississippi PTSD Scale; PTSD, post-traumatic stress disorder; PTSS, post-traumatic stress symptoms; SCL-90, Symptom Checklist-90.

**Table 3:** Studies assessing psychological consequences of the Fukushima nuclear disaster.

Reference/ Nuclear Disaster	Sample	Final Sample Size	Study Period	Outcomes	Key Findings
Anderson et al. (2016) [28]	Fukushima Medical University students	494 (132 volunteers, 362 non-volunteers)	July 2014	Post-traumatic growth, psychological distress (confusion, anger, and sadness)	Higher post-traumatic growth in volunteers than non-volunteers, but no difference in distressing symptoms.
Aoki et al. (2014) [29]	Clinical records of all patients who visited the Ohta Nishinouchi medical center in the study period	981 (493 in control year, 488 in study year)	March 2010–March 2011, March 2011–March 2012	Non-fatal suicide attempts	The risk of suicide attempt by high-mortality means was elevated for four months after the disaster. There was no change in rates of low lethality attempts.
Cavanagh et al. (2014) [30]	Members of the U.S. Embassy in Tokyo	120	July 2011	Psychological functioning (PTSS, depressive symptoms, and life satisfaction)	Self-reported use of cognitive reappraisal was not related to psychological functioning, but demonstrated success using cognitive reappraisal techniques was associated with fewer symptoms of depression and PTSS.
Goto et al. (2015) [31]	Women living in Fukushima who registered their pregnancies in a one-year period	8196	August 2010–July 2011	Depressive symptoms (two-item screening measure)	28% of women reported depressive symptoms. Living close to the reactor was associated with greater depressive symptoms.
Goto et al. (2017) [32]	Women living in Fukushima who registered their pregnancies in a two-year period	13,109 (6686 in 2012, 6423 in 2013)	August 2011–July 2012, August 2012–July 2013	Depressive symptoms (two-item screening measure)	25% of mothers reported depressive symptoms in 2012, and 24% reported depressive symptoms in 2013. Higher radiation concern was associated with depressive symptoms.
Hidaka et al. (2016) [33]	Fukushima decontamination workers	512	August–October 2013	Radiation anxiety	44.7% of decontamination workers reported radiation anxiety. Socially isolated workers reported more anxiety over radiation exposure.
Hori et al. (2016) [34]	New patients in Fukushima outpatient psychiatry clinics in a three-month period	2504 (771 in 2010, 1000 in 2011, 733 in 2012)	March–June 2010, March–June 2011, March–June 2012	Diagnosis of ASD, PTSD, adjustment disorder and depression (ICD-10)	Increased incidence of new patients with ASD and PTSD in 2011 and decreased incidence of new patients with depression. These results returned to pre-disaster levels in 2012.
Ikeda et al. (2017) [35]	Fukushima nuclear power plant workers at TEPCO Daiichi (affected reactor) and Daini (intact reactor)	1417 (1053 from Daiichi, 707 from Daini)	May–June 2011, May–June 2012, November 2013,November 2014	Psychological Distress (K6), PTSS (IES-R)	Post-traumatic stress response symptoms decreased over time but remained elevated three years after the nuclear disaster.
Imamura et al. (2016) [36]	Mothers with preschool children in Fukushima city and surrounding areas	37 (18 in behavioural activation intervention group, 19 in control group)	August 2014, September 2014, November 2014	Psychological distress (K6), physical symptoms (BJSQ), radiation anxiety, positive well-being (liveliness and life satisfaction)	Behavioural activation was associated with lower psychological distress and less physical symptoms at the one-month follow-up, but not at three months. Behavioural activation was associated with higher life satisfaction and increased liveliness at the three-month follow-up.
Ishii et al. (2017) [37]	Women who received Maternal and Child Health Handbooks from municipal offices in Fukushima from 2011 to 2014	60,860 (16,001 in 2011, 14,516 in 2011, 15,218 in 2013, 14,516 in 2014)	2011–2014	Depressive symptoms (two-item screening measure)	27% of mothers reported depressive symptoms in 2011, 26% in 2012, 25% in 2013, and 23% in 2014.
Ishikawa at al. (2015) [38]	Undergraduates from universities in Fukushima, Tokyo, and Kyoto	435 (106 from Fukushima, 176 from Tokyo, 153 from Kyoto)	September–December 2013	Trauma response (IES-R), depressive symptoms (CES-D), anger (STAXI), anxiety (SEA)	Tokyo undergraduates had the most significant traumatic response immediately after the earthquake. Fukushima undergraduates had the highest levels of anger. Kyoto undergraduates had more anxiety and depressive symptoms 2.5 years after the nuclear disaster than immediately after the accident.
Ito et al. (2018) [39]	Female college students	288	December 2015	Depressive symptoms (WHO-5), radiation risk perception	46.5% of female college students reported depressive symptoms. Higher radiation risk perception predicted reduced reproductive confidence, which was ultimately associated with increased depressive symptoms.
Kakamu et al. (2019) [40]	Radiation decontamination workers	531	August–October 2013	Type of anxiety	91.6% of decontamination workers reported at least one type of anxiety. Job security was the most common type of anxiety (41.8%) and working hours was the least common (6.0%).
Kashiwazaki et al. (2020) [41]	Residents of Fukushima and Tokyo aged 20–59 years	832 (416 from Fukushima, 416 on Tokyo)	August 2018	Health anxiety (HAI), psychological distress (K6)	Greater health anxiety was associated with more psychological distress. Mindfulness was associated with lower health anxiety and less psychological distress.
Kawakami et al. (2020) [42]	Adults living in temporary housing for three years after the nuclear disaster and a control group of residents from non-disaster areas of East Japan	1941 (1089 in shelter group, 852 in control group)	June–August 2014	MDE, manic or hypomanic episode, GAD, panic disorder, PTSD, and alcohol use disorder (CIDI)	The shelter group had a higher incidence of new mood and anxiety disorders in the first year after the disaster, but not in subsequent years. The remission rate for mood and anxiety disorders was lower in the shelter group.
Kohzaki et al. (2015) [43]	Citizens, doctors, and medical students inside and outside Fukushima	2487 (1557 in 2011, 930 in 2013)	September–October 2011; August–November 2013	Radiation anxiety	Citizens living in Fukushima were more anxious than those living outside Fukushima. Medical students who recently studied radiation biology were less anxious than the other groups. All three groups reported dissatisfaction with the government and TEPCO after the nuclear accident.
Kukihara et al. (2014) [44]	Evacuees from Hirono Town	241 (116 men, 125 women)	December 2011	PTSS (IES-R), depressive symptoms (ZSDS), resilience (CD-RISC)	53.5% reported symptoms of PTSD, and 66.8% reported symptoms of depression. Resilience was shown to be a protective factor for PTSD, depression, and general health.
Kuroda et al. (2017) [45]	Elderly evacuees without a baseline depressive tendency	438	May 2010, May 2013	Depressive tendency (BCL)	In elderly evacuees who did not report a depressive tendency at baseline, 37.2% had a depressive tendency at the second survey. Depressive tendency was associated with female sex, older age, and less engagement in social activities.
Kuroda, Iwasa, Orui, Moriyama, Nakayama, and Yasumura (2018) [13]	Fukushima residents	777 (606 from non-evacuation areas, 171 from evacuation areas)	August–October 2016	Radiation anxiety, discrimination and prejudice based on radiation exposure	Higher health literacy was associated with lower radiation anxiety in both areas and associated with lower discrimination and prejudice in the evacuation areas.
Kusama et al. (2018) [46]	Residents of Japan	10,000	March 2012	Anxiety, radiation risk-averse behaviours	23.0% of participants reported anxiety and 12.0% engaged in radiation risk-averse behaviours. Those with higher socioeconomic status felt less anxious, but engaged in more risk-averse behaviours than those with lower socioeconomic status.
Lebowitz (2016) [47]	Residents from Hirakata, Japan, and Otsu, Japan	466 (351 female, 115 male)	December 2011–March 2012	Depression (CES-D)	23% of female participants and 17% of male participants met criteria for depression. The strongest predictors of depression were property damage and younger age.
Lebowitz (2017) [48]	Residents from Hirakata, Japan, and Otsu, Japan	466 (351 female, 115 male)	December 2011–March 2012	Depression (CES-D)	Relational satisfaction from both providing and receiving social support buffers against depression.
Maeda et al. (2016) [49]	Fukushima public employees working in two coastal towns that were initially evacuated	168 (92 from Town A where evacuation restrictions were lifted several months after the accident, 76 from Town B where evacuation orders remained at time of study)	March–October 2013	Depression, PTSD, and suicide risk (MINI)	17.9% of public employees met criteria for depression, and 4.8% met criteria for PTSD. 8.9% screened positive for suicide risk.
Murakami et al. (2017) [14]	Residents of Marumori Town, Japan	174	March 2015	Radiation anxiety, perceptions of radiation risk, well-being	Higher evaluation of the town’s decontamination efforts was associated with a reduction in radiation anxiety.
Murakami, Hirosaki et al. (2018) [50]	Fukushima evacuees	34.312	2011–2012	Frequency of laughter, mental health distress (K6), radiation anxiety	Laughing more frequently was associated with lower radiation anxiety in the absence of mental health distress, but not in the presence of mental health distress.
Murakami, Takebayashi et al. (2018) [51]	Fukushimaresidents	1023	August 2016	Radiation anxiety, well-being	Certain radiation countermeasures were associated with lower well-being (thyroid exam, food inspection, explanatory meetings), but the basic survey was associated with greater well-being. The thyroid exam is associated with less radiation anxiety.
Nagamine et al. (2018) [52]	Japan Ground Self-Defense Force personnel deployed to the Great East Japan Earthquake	56,753	1, 6, and 12 months post-mission completion of deployment	PTSS (IES-R), psychological distress (K10)	Duties with radiation exposure risk were not associated with PTSS or psychological distress.
Nakayama et al. (2019) [15]	Fukushima residents	868	August 2016	Radiation anxiety	Radiation anxiety was higher for people who utilized internet sources for information about the nuclear disaster and lower for people who utilized local broadcast TV. Radiation anxiety was lower for people who trusted government sources of information and higher for people who trusted citizen groups.
Oe, Fujii et al. (2016) [53]	Fukushima residents living in evacuation zones	169,175 (71,100 in January 2012, 53,162 in January 2013, 44,913 in February 2014)	January 2012, January 2013, February 2014	Psychological distress (K6), PTSS (PCL)	Prevalence of PTSS for men was 18.6% in 2012, 16.3% in 2013, and 15.0% in 2013. Prevalence of PTSS for women was 24.9% in 2012, 19.9% in 2013, and 18.1% in 2014.
Oe, Maeda et al. (2016) [54]	Fukushima residents living in areas that were considered complete evacuation zones for three years after the disaster	12,371	2011, 2012, 2013	Psychological distress (K6), radiation risk perception	Higher psychological distress was associated with greater radiation risk perception and poor social support.
Oe et al. (2017) [55]	Fukushima residents living in areas that were considered complete evacuation zones for three years after the disaster	12,371	2011, 2012, 2013	PTSS (PCL), radiation risk perception	Four trajectories of PTSS were demonstrated: PTSS trajectories: chronic (8.1%), resistant (54.9%), recovered (19.3%), and non-recovered (17.7%).
Orui et al. (2020) [16]	Fukushima residents	225 (156 forced evacuees, 69 voluntary evacuees)	August–October 2016	Radiation anxiety	Use of public relations information from local government was associated with lower anxiety for forced evacuees, but not voluntary evacuees.
Orui et al. (2018) [56]	Vital statistics from the Ministry of Health, Labour, and Welfare on suicide rates in Japan during the study period	n/a	March 2009–December 2015	Monthly suicide rate	Male suicide rates in evacuation areas increased immediately after the nuclear disaster, then increased again four years after the disaster. Overall, suicide rates decreased for males 50–69 years, but increased for males younger than 30 and 70 and older. Female suicide rates declined during the first year and then increased over the next three years.
Rubin et al. (2012) [57]	British nationals in Japan	284	December 2011	Psychological distress (GHQ-12), anger (STAXI-2), anxiety (STAI)	16% reported psychological distress, 29.7% reported anxiety, and 30.4% reported anger. Utilizing low credibility sources was associated with greater distress, anger, and anxiety.
Shigemura et al. (2018) [58]	Male dentists who conducted disaster victim identification (DVI) in Fukushima after the 2011 disaster	49	September-December 2011	Psychological distress (GHQ-12), PTSS (IES-R)	Greater psychological distress was associated with younger age and property loss. PTSS was associated with extensive property loss.
Shigemura et al. (2014) [59]	Fukushima nuclear power plant workers at TEPCO Daiichi (affected reactor) and Daini (intact reactor)	1411 (831 from Daiichi, 580 from Daini)	May–June 2011	PTSS (IES-R)	For both plants, PTSS was highly associated with peritraumatic distress. Experiencing discrimination and the presence of a pre-existing illness were also associated with PTSS.
Shigemura et al. (2020) [10]	Systematic review of studies on the psychological consequences of the Fukushima disaster	79 studies	August 2019	Psychological distress, PTSS, anxiety	Rates of psychological distress ranged from 8.3 to 65.1%. Rates of depressive symptoms ranged from 12 to 52.0%. Rates of PTSS ranged from 10.5 to 62.6%.
Sugimoto et al. (2013) [17]	Fukushima residents	969	June–July 2011	Radiation anxiety	Utilizing rumours as a source of information about the disaster increased radiation anxiety. Attending a seminar on radiation reduced radiation anxiety.
Takebayashi et al. (2017) [12]	Systematic review of studies on risk perception and anxiety regarding radiation among people living in Japan after the 2011 Fukushima nuclear disaster	24 studies	May 2017	Radiation anxiety	Radiation anxiety is associated with demographics, disaster-related stressors, trusted information, and radiation-related stressors.
Tanisho et al. (2016) [60]	Fukushima nuclear power plant workers at TEPCO Daiichi (affected reactor) and Daini (intact reactor)	968 (571 from Daiichi, 397 from Daini)	May–June 2011, May–June 2012	Psychological distress (K6), PTSS (IES-R)	Experiencing discrimination at time one predicted higher psychological distress and PTSS at time two. Higher PTSS at time one predicted higher PTSS at time two. PTSS was associated with older age.
Terayama et al. (2020) [11]	Systematic review of studies on the emotional and behavioural consequences of the 2011 Fukushima nuclear disaster	61 studies	August 2019	Emotional and behavioural consequences of the Fukushima nuclear disaster	Radiation risk perception was associated with immediate health effects and fear of future health effects. Survivors of nuclear disasters experience lower well-being, greater discrimination, and have an increased rate of suicide.
Tsubokura et al. (2014) [61]	Fukushima residents of Iitate village and Soma city who underwent annual health evaluations in the year before and the year after the disaster	564	May 2011	Depressive symptoms (PHQ-9)	12% of participants met criteria for depression.
Tsujiuchi et al. (2016) [62]	Fukushima evacuees living in Saitama prefecture	350	March–April 2012	PTSS (IES-R)	59.4% of participants had symptoms consistent with a diagnosis of PTSD. Predictors of PTSD included chronic physical and mental illness, lost jobs, and limited social support.

Abbreviations: ASD, Acute Stress Disorder; BCL, Basic Checklist; BJSQ, Brief Job Stress Questionnaire; CD-RISC, Connor-Davidson Resilience Scale; CES-D, Center for Epidemiologic Studies Depression Scale; CIDI, Composite International Diagnostic Interview; GHQ-12, General Health Questionnaire-12; HAI, Health Anxiety Index; ICD-10, International Classification of Diseases, 10th edition; IES-R, Impact of Event Scale-Revised; K6, Kessler 6-Item Psychological Distress Scale; K10, Kessler 10-Item Psychological Distress Scale; MDE, major depressive episode; MINI, Mini-International Neuropsychiatric Interview; n/a, not available; PCL, PTSD Checklist; PHQ-9, Patient Health Questionnaire-9; PTSD, post-traumatic stress disorder; PTSS, post-traumatic stress symptoms; SEA, Spence–Essau Anxiety Questionnaire; STAI, State-Trait Anxiety Inventory; STAXI, State-Trait Anger Expression Inventory; STAXI-2, State-Trait Anger Expression Inventory 2; TEPCO, Tokyo Electric Power Company; WHO-5, World Health Organization-Five Well-Being Index; ZSDS, Zung Self-Rating Depression Scale.

## Data Availability

Not applicable.
